# Atypical manifestations of recent syphilis: study of 19 cases^☆^^☆☆^

**DOI:** 10.1016/j.abd.2020.03.008

**Published:** 2020-07-15

**Authors:** José Carlos Sardinha, Livia Lima de Lima, Marcel Heibel, Antonio Schettini, Sinesio Talhari, Carolina Talhari

**Affiliations:** a Department of Sexually Transmitted Infections, Fundação Alfredo da Matta de Dermatologia e Venereologia, Manaus, AM, Brazil; b Department of Urology, Universidade do Estado do Amazonas, Manaus, AM, Brazil; c Department of Dermatopathology, Fundação Alfredo da Matta de Dermatologia e Venereologia, Manaus, AM, Brazil; d Department of Dermatology, Universidade do Estado do Amazonas, Manaus, AM, Brazil

**Keywords:** Syphilis, Syphilis, cutaneous, Treponema pallidum, Treponemal infections

## Abstract

**Background:**

Syphilis is one of the most common diseases that start with genital ulcers. Aside from the initial, classic ulcerative lesion of syphilis, called hard chancre, atypical presentations are common, with erosions, erythema, edema, balanitis, and other dermatological manifestations. Associated with initial genital lesions, the presence of inguinal adenopathies is frequent, and the presence of hardened and painless lymphangitis on the dorsum of the penis is rare.

**Objectives:**

To describe atypical penile manifestations in patients with early syphilis.

**Methods:**

The present study reports patients who developed cord-like lesions on the penis.

**Results:**

The study included 25 patients with cord-like lesions on the penis; in 19 of those, the diagnosis of syphilis was confirmed.

**Study limitations:**

Small number of patients included.

**Conclusions:**

In view of the findings of the present investigation, it is important to emphasize that all patients who present with cord-like lesions on the penis must undergo a rapid test for syphilis, VDRL, serologies for HIV viral hepatitis B and C and, whenever possible, histopathological and Doppler exams.

## Introduction

Sexually Transmitted Infections (STIs) are, in general, characterized by vaginal discharge, urethral discharge, verrucous lesions, vegetating lesions, or genital ulcerations.^1^, ^2^, ^3^

Genital ulcers are common and can be suggestive of certain diseases or they can be atypical and difficult to diagnose.^1^, ^2^, ^4^

Syphilis is one of the most common diseases that start with genital ulcers. Aside from the initial, classic ulcerative lesion of syphilis, called hard chancre, atypical presentations are common, with erosions, erythema, edema, balanitis, and other dermatological conditions.^1^, ^3^, ^4^ Associated with initial genital lesions, the presence of inguinal adenopathies is frequent, and the presence of hardened and painless lymphangitis on the dorsum of the penis is rare.^5^

Among the various clinical manifestations of syphilis, the authors of the present study have observed, in patients with recent syphilis, painless cord-like lesions, hardened on palpation, located mainly in the balanopreputial groove.^6^ Similar lesions have also been observed in patients for whom a definitive conclusion about the etiology of the dermatological manifestation can not be obtained.

The present study reports 25 patients with cord-like lesions on the penis; in 19 of those, the diagnosis of syphilis was confirmed.

## Methods

In this retrospective study, 25 patients with clinical suspicion of recent syphilis, presenting lesions with a cord-like aspect in the balanopreputial groove, had their medical records evaluated. These patients were treated at an STI diagnosis and treatment center, from January 2015 to February 2018. Demographic data, history of sexual activities, clinical evolution of the manifestations observed, and results of laboratory tests were surveyed.

All 25 patients underwent rapid test (treponemic), immunochromatographic testing (SD 65, Korea), and the venereal disease research laboratory panel (VDRL, Laborclin ― Brazil), as a non-treponemic test. Furthermore, as routine exams, a rapid immunochromatographic test for HIV (Quibasa – BH/MG, Brazil) and serology for hepatitis B (BioMerieux ― France) and C (SD 65, Korea) were performed.

In six patients, biopsies were performed (with 3 or 4 mm punches) for histopathological, immunohistochemical, and polymerase chain reaction (PCR) assessments. The sections for histopathological examination were stained with hematoxylin-eosin. Immunohistochemistry examination was performed in two patients, using anti-Treponema pallidum monoclonal antibodies (Dako ― Santa Clara, CA, United States). PCR was performed in four patients. To perform the PCR, probes and primers developed in the molecular biology laboratory of the center where the study was developed were used, targeting the polA gene of T. pallidum, adapted from Leslie et al.^7^

This study was approved by the Ethics Committee of the STI diagnosis and treatment center where the data were collected (CAAE: 13217519.0.0000.0002). All patients signed an informed consent form to participate in the study.

## Results

The age of the 25 patients ranged from 19 to 65 years, with a mean of 28.6 years and a median of 26 years.

The patient with the longest clinical evolution reported the presence of the disease for 150 days; he presented repeated negative syphilis tests (rapid test and VDRL). In other cases, the clinical picture ranged from 3 to 30 days of evolution.

**Figure 1:**
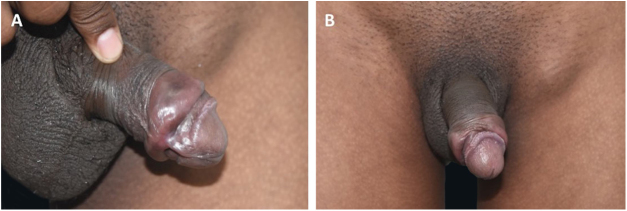
(A) Recent syphilis. Cord-like lesion (VDRL 1:128). (B), Notice the large-volume painless adenomegaly.

**Figure 2:**
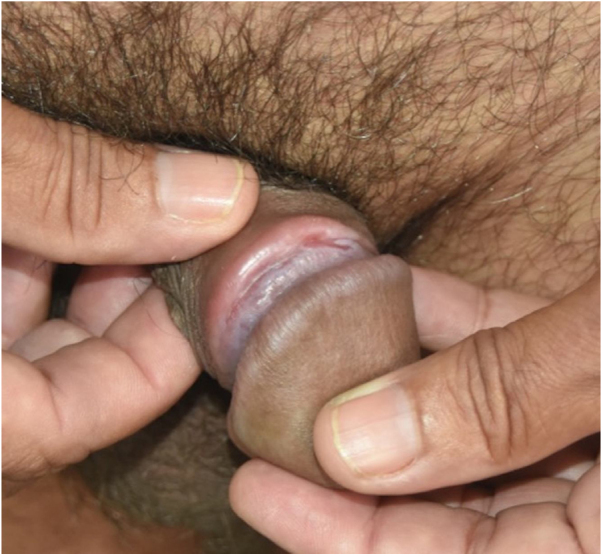
Recent syphilis. Cord-like lesion and shallow, clean ulcer.

**Figure 3:**
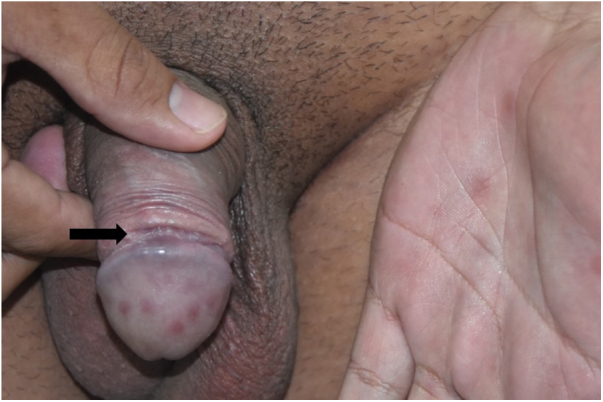
Cord-like lesion and underlying shallow ulcer along almost the entire indurated area. Notice the secondary lesions in the hand and glans.

All patients had lesions with arciform disposition, of cartilaginous consistency on palpation, of variable sizes, located around the balanopreputial groove (Figs. 1A and 1B, 2A and 2B and 3). In some patients, mild ulcerations and scars were observed in the vicinity of the hardened areas (Figs. 1A and 1B, and 3). Two (8%) patients presented inguinal lymphadenomegaly and syphilitic roseola, associated with penile lesions (Figure 2, Figure 3).

The VDRL was positive in 19 patients (76%), with variable titrations from 1:4 to 1:512 (mode: 1:32). In all patients with reagent VDRL, the rapid treponemic test was also positive. No pattern was identified between the time of evolution and the positivity for the rapid test, or between the time of evolution and VDRL titration.

HIV serology was positive in two patients (8%); one (4%) of them was on regular antiretroviral treatment. Serological tests for viral hepatitis B and C were negative in all patients.

The six patients (24%) with negative complementary tests for syphilis were clinically similar to those with confirmed treponematosis. The probable causes, in two cases (33.3%), were balanitis associated with psoriasis and seborrheic dermatitis. In the other four patients (66.6%), no inflammatory processes were observed in the glans or balanopreputial groove.

**Figure 4:**
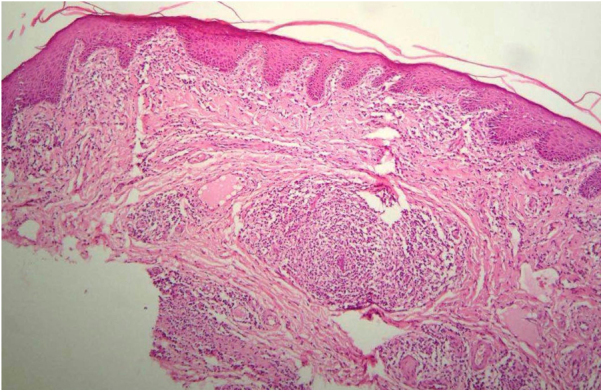
Hyperkeratosis and regular acanthosis of the epithelium. In the chorion, lymphoplasmacytic infiltrate surrounding superficial vessels (Hematoxylin & eosin, ×100).

Histopathological examination of the six patients (31%) whose penile lesions were biopsied showed alterations usually observed in recent syphilis lesions: epidermis with hyperkeratosis and acanthosis; in the dermis, the infiltrate varied from mild to moderate, arranged around the vessels, with tumefied endothelial cells (Fig. 4). Two patients (10.5%) presented ill defined granulomas, formed by histiocytes, lymphocytes, and rare epithelioid cells. In five cases (26.3%) numerous plasma cells were found and two (10.5%) patients presented neutrophils in the infiltrate. In these six cases (31.5%), the diagnosis of syphilis was confirmed.

**Figure 5:**
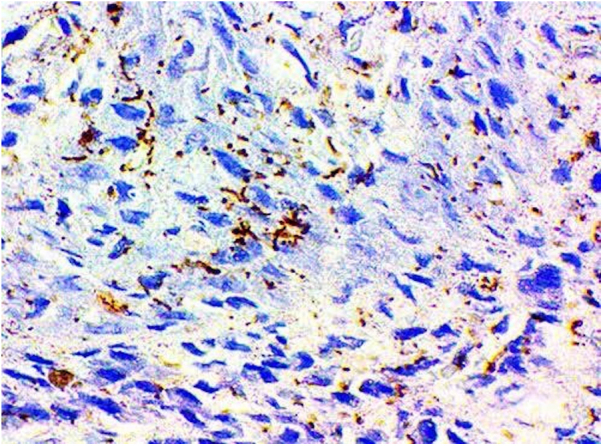
Immunohistochemistry − Patient # 1. Positive for *Treponema pallidum*.

PCR for T. pallidum was positive in all four cases in which this test was performed. The presence of treponema was demonstrated in the two patients who underwent immunohistochemistry (Fig. 5). In all of these cases, there was agreement with the positive serological results for syphilis.

The 19 patients diagnosed with syphilis were classified as having recent infection and treated with a single dose of 2,400,000 IU of penicillin G benzathine, according to the recommendation of the Brazilian Ministry of Health.^3^ All patients presented clinical cure, with complete lesion resolution within 30 days; they continue to be regularly followed-up, with clinical evaluation and control serology for syphilis every three months.

## Discussion

STIs, and particularly syphilis, are an important global public health issue. The increase in the number of syphilis cases has been significant in recent years, affecting populations of all socioeconomic levels.^3^ In Brazil, there was an increase from 2.0 cases/100,000 inhabitants, in 2010, to 42.5/100,000 inhabitants, in 2016. It is important to note that syphilis notification became mandatory in Brazil in 2010. Therefore, this increase in cases may also be related to the better records.^3^

Over the last four decades, the professionals at the reference center where the study was carried out met an important demand for STI treatment in the state capital where the center is located. From 2010 to 2018, 4,373 new cases of syphilis were observed in this center.

The clinical presentations of syphilis, in all phases, are varied, and it is considered to be “the great imitator” by most scholars in this area. In the early phase, mainly in the genital region, the initial lesions of syphilis can present the typical, classic ulcerative aspect, called hard chancre. However, in many cases, the clinical aspect may be non-specific, or atypical.

In the present study, 25 patients with hardened lesions, in arciform disposition, located around the balanopreputial groove, similar to Mondor’s disease (MD) in an atypical location, are reported.^8^, ^9^ Among these 25 patients, 19 had a recent clinical and laboratory diagnosis of syphilis. Six of these 19 cases presented an evolution that ranged from 3 to 30 days, with negative serology at the first consultation, and positive after one week. The literature indicates that treponemal tests can be negative in 30% of cases at the first consultation of patients with lesions suggestive of primary syphilis.^10^

In the six cases in which the histopathological examination was performed, an inflammatory infiltrate suggestive of recent infection was found; all of these patients had laboratory confirmation for syphilis.

Among the references consulted, current and classic, only Fournier, in 1889,^17^ described recent syphilis with a cord-like aspect.^1^, ^2^, ^11^, ^12^, ^13^, ^14^, ^15^, ^16^, ^17^

According to one of the authors of this article, no cases of penile cord-like lesion in recent syphilis were diagnosed in the center where the study was conducted in the period prior to 2015. It is possible that the increased numbers of patients with STIs, in the center where the patients were included, and, consequently, of the cases of syphilis and atypical penile lesions, are related to the restrictive measures for the purchase of antibiotics, established in Brazil as of 2010 (Resolution RDC 44, of October 26, 2010, from the Brazilian National Health Surveillance Agency [ANVISA]).

In the differential diagnosis of penile lesions with the clinical aspects presented in this study, mainly MD and sclerosing lymphangitis (SL) should be considered.^8^, ^9^, ^18^, ^19^, ^20^, ^21^, ^22^

The MD, described in 1939, is secondary to superficial thrombophlebitis, with a typical location on the chest wall.^20^ Penile MD is secondary to superficial thrombophlebitis of the dorsal vein of the penis; it is painful on palpation and the diagnosis is relatively easy through Doppler ultrasonography. Vascular impairment may be associated with excessive sexual activity, intense physical exercise, or malignant neoplasias. Depending on the time of evolution, histopathological examination may be necessary; it usually evidences thrombus formation, recanalization, fibrosis, and thickening of the venous wall.^8^, ^9^, ^18^, ^19^, ^20^, ^21^ In 2009, a case of MD associated with primary syphilis was reported.^8^

For most authors, SL is not of venereal etiology as well, being practically indistinguishable from MD.^21^ It may be associated with intense sexual practice, and there are references of its association with circumcision. There is disagreement regarding the pathogenesis of SL; impairment of lymphatic and venous vessels has already been reported. In the histopathological examination of SL, there is thickening of the lymph vessel wall, with partial or total lumen occlusion and, occasionally, chronic inflammatory process.^22^ Although penile SL and MD are rarely of venereal etiology, routine STI exams are suggested in all cases with this topography.^8^, ^9^, ^18^, ^19^, ^20^, ^21^, ^22^ In all patients in the present study, there was no involvement of the dorsal vein of the penis. The lesions were arciform, around the balanopreputial groove.

Some patients in this case series had lesions similar to “giant syphilitic chancre”, characterized by the presence of ulcers, associated with cord-like lesions, located in the balanopreputial groove. These patients also had multiple erosions of treponematous origin, located in the glans, a picture called syphilitic balanitis of Follmann (SBF).^23^ Mainetti et al. reported five cases of SBF, and one of the patients also presented a hardened, cord-like area.^24^ In the cases of syphilis in the present study, the ulcerations and cicatricial areas were discreet, almost imperceptible or non-existent in most of the 19 cases with a confirmed diagnosis of syphilis (Figure 2).

In a more recent publication, under the name “Indurative edema of the foreskin simulating phimosis, an atypical manifestation of primary syphilis,” two cases with lesions similar to the ones described in the present investigation are described, called “necklace lesions.”^8^ In this study, the authors draw attention to the differential diagnosis between syphilitic phimosis, characterized by fibrosis of the foreskin, and hardened edema, a complication of hard chancre located in the coronal sulcus or internal surface of the foreskin. Despite the hardened, necklace-like appearance observed in the 19 cases in the present study, none progressed to syphilitic phimosis.

A clinical picture similar to that of the 19 syphilis cases described in the present study is shown in a photo of the STI chapter of a classic textbook, being described as SL.^11^ The author however does not present diagnostic evidence.

It is noteworthy that cord-like lesions, similar to those observed in patients with syphilis in the present study, may be related to other inflammatory processes of the glans. In six of the 25 patients studied, serology for syphilis was negative, and it is reasonable to consider a probable association with balanitis secondary to psoriasis, seborrheic dermatitis, chronic ulceration of the glans, and unidentified causes. To date, there is no explanation for the pathophysiology of the manifestations observed in these patients.

## Conclusions

In light of the findings of the present investigation, it is important to emphasize that all patients who present with cord-like lesions in the penis must undergo a rapid test for syphilis, VDRL, serologies for HIV and viral hepatitis B and C and, whenever possible, histopathological and Doppler exams.

## Financial support

None declared.

## Authors’ contributions

José Carlos Sardinha: Approval of the final version of the manuscript; conception and planning of the study; elaboration and writing of the manuscript; obtaining, analyzing, and interpreting the data; critical review of the manuscript.

Livia Lima de Lima: Elaboration and writing of the manuscript; critical review of the literature; critical review of the manuscript.

Marcel Heibel: Conception and planning of the study; elaboration and writing of the manuscript; obtaining, analyzing, and interpreting the data; intellectual participation in propaedeutic and/or therapeutic conduct of studied cases.

Antonio Schettini: Approval of the final version of the manuscript; obtaining, analyzing, and interpreting the data; effective participation in research orientation; intellectual participation in propaedeutic and/or therapeutic conduct of studied cases; critical review of the manuscript.

Sinesio Talhari: Approval of the final version of the manuscript; conception and planning of the study; elaboration and writing of the manuscript; obtaining, analyzing, and interpreting the data; effective participation in research orientation; critical review of the manuscript.

Carolina Talhari: Approval of the final version of the manuscript; conception and planning of the study; elaboration and writing of the manuscript; obtaining, analyzing, and interpreting the data; effective participation in research orientation; intellectual participation in propaedeutic and/or therapeutic conduct of studied cases; critical review of the literature; critical review of the manuscript.

## Conflicts of interest

None declared.

## References

[1] Belda W., IBelda W., Chiacchio N., Criado P.R. (2010). Sífilis Adquirida e Congênita. Tratado de Dermatologia.

[2] Sampaio S.A.P., Rivitti E.A. (2018). Dermatologia.

[3] (2017). Ministério da Saúde. Manual Técnico para Diagnóstico da Sífilis.

[4] Degos R. (1981). Muqueuse Génitale Masculine - Syphilis primaire. Traité de Dermatologie. France: Flammarion Médicine-Sciences.

[5] Rani R. (2009). Mondor’s disease of the penis associated with primary syphilis. Int J STD AIDS..

[6] Sardinha J.C.G., Ramos M.C., Schettini A.P.M., Talhari S. (2018). Case for diagnosis. Atypical genital lesion. An Bras Dermatol..

[7] Leslie D.E., Azzato F., Karapanagiotidis L., Leydon J., Fyfe J. (2007). Development of a real-time PCR assay to detect Treponema pallidum in clinical specimes and assessment of the assay’s performance by comparison with serological testing. J Clin Microbiolol..

[8] Vartolomei M.D., Cotoi O.S., Badea M.A., Chibelean C.B., Cotoi T., Morariu V. (2015). Indurative Edema of the Prepuce Mimicking Phimosis, an Atypical Manifestation of Primary Syphilis. Acta Dermatovenereol Croat..

[9] Arora R., Sonthalia S., Gera T., Sarkar R. (2015). Atypical Penile Mondor’s Disease − Involvement of the Circumflex Vein. Int J Std Aids..

[10] Larsen S., Steiner B., Rudolph A. (1995). Laboratory diagnosis and interpretation of tests for syphilis. These include: Laboratory Diagnosis and Interpretation of Tests for Syphilis. Clin Microbiol Rev..

[11] Burns T, Breathnach S., Cox N., Griffiths Christopher (2013). Rook’s Textbook of Dermatology.

[12] Johnson R.A., Fitzpatrick TB, Eisen AZ, Wollf K, Freedberg IM, Austen KF (1999). Diseases and disorders of the anogenitalia of males. Dermatology In General Medicine.

[13] Braun-Falco O., Plewig G., Wolff H.H., Burgdorf W.H.C. (1996). Syphilis. Dermatology.

[14] Bechelli L.M., Curban G.V. (1975). Treponematoses. In: Compêndio de Dermatologia.

[15] Hutchinson J. (1887). Syphilis.

[16] Gaucher P.C.E. (1907). Le chancre et les syphilides cutanees et muqueuses et le traitement general de la syphilis/par E. Gaucher … Enseignement clinique de l’Hopital Saint-Louis recueilli par le dr Lacapere.

[17] Fournier A. (1898). Variétes Du Chancre Chez L Homme. Traité de la Syphilis.

[18] Öztürk H. (2014). Penile Mondor’s disease. Basic Clin Androl.

[19] Boscolo-Berto R., Raduazzo D.I. (2012). Penile Mondor’s disease: Long-term functional follow-up. Urol J.

[20] Walsh J.C., Poimboeuf S., Garvin D.S. (2014). A common presentation to an uncommon disease. Penile Mondor’s disease : A case report and literature review. Int Med Case Rep J.

[21] Barseló E.R., Martín J.A.P., Gomez M.C., Baños J.L.G., Tubet C.A., Diego R.B. (2008). Enfermedad de Mondor versus linfangitis esclerosante de pene. Arch Esp Uro.

[22] Babu A.K., Krishnan P., Andezuth D.D. (2014). Sclerosing lymphangitis of penis- literature review and report of 2 cases. Dermatol Online J.

[23] Lejman K., Starzycki Z. (1975). Syphilitic balanitis of Follmann developing after the appearance of the primary chancre − a case report. Brit J Vener Dis.

[24] Mainetti C., Scolari F., Lautenschlager S. (2016). The clinical spectrum of syphilitic balanitis of Follmann: report of five cases and a review of the literature. J Eur Acad Dermatol Venereol.

